# Effects of world knowledge on the prediction of upcoming verbs: an eye-tracking study

**DOI:** 10.1007/s10936-022-09900-9

**Published:** 2022-07-05

**Authors:** Juan Vela-Candelas, Natàlia Català, Josep Demestre

**Affiliations:** 1 Department of Psychology and CRAMC, Universitat Rovira i Virgili, Tarragona, Spain; 2 Department of Romance Studies, Universitat Rovira i Virgili, Tarragona, Spain; 3 Departament de Psicologia, Universitat Rovira i Virgili, Carretera de Valls, s/n, 43007 Tarragona, Spain

**Keywords:** World knowledge, Selectional restrictions, Event knowledge, Eye-tracking, Sentence comprehension

## Abstract

Some theories of sentence processing make a distinction between two kinds of meaning: a linguistic meaning encoded at the lexicon (i.e., selectional restrictions), and an extralinguistic knowledge derived from our everyday experiences (i.e., world knowledge). According to such theories, the former meaning is privileged over the latter in terms of the time-course of its access and influence during on-line language comprehension. The present study aims to examine whether world knowledge anomalies (that do not violate selectional restrictions) are rapidly detected during online sentence processing. In an eye-tracking experiment, we used materials in which the likelihood of a specific verb (*entrevistar* or *secuestrar*, the Spanish translations for *to interview* and *to kidnap*) depended on the agent of the event (*periodista* or *terrorista*, the Spanish translations for *journalist* and *terrorist*). The results showed an effect of typicality in regression path duration and total reading times at both the verb region and the spillover region, thus providing evidence that world knowledge is rapidly accessed and used during on-line sentence comprehension.

## Introduction

People build up an enormous amount of knowledge about how the world is and how it works through everyday experience. This knowledge plays an important role in social interactions and may also influence language comprehension and production. Daily conversations normally involve talking about what we have done, what we are doing or what we will do in the future. In order to convey all this information in an appropriate manner, we turn to our knowledge about common events (e.g., going to a restaurant) and the typical elements that take part in them (e.g., the waiter, the meal, the bill and so on). This also allows us to omit some details that we assume our interlocutor infers because of our shared knowledge of the world.

In line with these intuitively obvious ideas, all theories of sentence processing agree in considering that common world knowledge somehow influences our ability to comprehend language. This is nothing but a logical conclusion: if the human mind seems to be able to use different types of information (e.g., phonological, semantic, syntactic, pragmatic), why should it renounce profiting from one of them? Therefore, the core differences across sentence processing theories lie in how and when this kind of extralinguistic knowledge is accessed and used during online comprehension.

According to some theories, it is possible to make a distinction between two kinds of meaning: a linguistically relevant meaning (i.e., linguistic semantics), and an extralinguistic and more pragmatic knowledge derived from our daily experiences (i.e., world knowledge). According to these accounts, during the first stage of real-time processing of a sentence, the processor has only access to linguistically relevant information, and only later on it can access and use other types of information (Bornkessel & Schlesewsky, 2006; Fodor, 1983; Frazier & Clifton, 1996; Frazier & Fodor, 1978). The question that immediately arises is the following: what is meant by *linguistically relevant*? Many linguists argue that the key difference is that linguistically relevant meaning is lexically encoded and is part of the language system, while world knowledge is part of a comprehender’s general knowledge (Chomsky, 1975; Katz & Fodor, 1963; Sperber & Wilson, 1986). That would be the reason why the former is sometimes claimed to be privileged over the latter in terms of the time-course of its access and influence during sentence processing.

A crucial theoretical notion is that of selectional restrictions. Selectional restrictions, which are assumed to be part of the lexical entries stored in our mental lexicon, refer to the semantic constraints that a verb places on its arguments. For instance, the verb *to eat* selects an agent argument (the entity that performs the action expressed by the verb) that has to be animate, and a patient argument (the entity or object on which the action is performed) that has to be edible. The violation of any of these semantic constraints results in clearly anomalous sentences. However, there is no clear consensus on the nature of these restrictions. For some, they deal with semantic constraints (Brockmann & Lapata, 2003; Carnie, 2006); for others, they are basically syntactic in nature (Bornkessel & Schlesewsky, 2006). However, regardless the syntactic or semantic nature of selectional restrictions, they are usually considered as a key component of a verb’s lexical information. Thus, as lexically encoded information, selectional restrictions have played a very important role in interpreting the results of many sentence comprehension studies (see, for example, Altmann & Kamide 1999; Trueswell & Tanenhaus, 1994; Warren & McConnell, 2007).

In clear contrast, other scholars state that world knowledge can immediately influence online sentence comprehension (Chwilla & Kolk, 2005; Ferretti et al., 2001; Hagoort et al., 2004; Matsuki et al., 2011), some of them even question the possibility of establishing a sharp distinction between linguistic semantics and world knowledge (Jackendoff, 2002; Langacker, 2008), and some define selectional restrictions as abstractions computed over world knowledge (McRae & Matsuki, 2009).

The purpose of the present study is to further investigate whether there exists such a significant delay in the use of our common knowledge about events in comparison to a privileged status of lexical knowledge. In doing so, we focus on recent empirical studies that have reported evidence both supporting (Patson & Warren, 2010; Staub et al., 2007; Warren & McConnell, 2007) and rejecting (Bicknell et al., 2010; Chwilla & Kolk, 2005; Dickey & Warren, 2015; Hare et al., 2009a; Matsuki et al., 2011; McRae & Matsuki, 2009; Metusalem et al., 2012; Milburn et al., 2015) that processing may be different for lexical information versus world knowledge.

## Previous studies

Warren & McConnell (2007) conducted an eye tracking study testing reading in three experimental conditions: (1a) possible and plausible sentences, (1b) possible but implausible sentences –strange sentences in which no selectional restriction was violated–, and (1c) sentences that were impossible, as they included selectional restrictions violations:

- The man used a strainer to drain the thin spaghetti yesterday evening.
- The man used a blow-dryer to dry the thin spaghetti yesterday evening.
- The man used a photo to blackmail the thin spaghetti yesterday evening.

The authors found an immediate effect of impossibility at the patient of the non-finite verb, with longer first fixation durations for condition (1c). Thus, when there is a selectional restriction violation, immediate effects arise. However, what is critical here is that the effects for the plausible-implausible comparison were somewhat delayed. Warren & McConnell (2007, p. 774) concluded that “information about a verb’s selectional restrictions is privileged over other kinds of knowledge in comprehension.”

Nevertheless, during the last decade an increasing number of experimental studies have reported evidence that event-based knowledge is quickly accessed and used during online language comprehension. These investigations have explored the phenomenon in different ways. Some have focused on the activation of world knowledge at the word level. For instance, Ferretti et al. (2001) showed by using a lexical priming task that reading a certain verb facilitates the processing of nouns that refer to entities typically related to the event denoted by the verb (i.e., *arrest* primes *police*, *thief*, *handcuffs*…). According to McRae et al. (2005), the same is true the other way around, that is, nouns referring to entities that typically participate in a certain event prime the processing of the verb referring to this specific event (i.e., *handcuffs* prime *arrest*). The basic idea underlying these studies is that thematic role assignment is essentially based on our experiential event knowledge that can be activated by individual verbs and nouns, and not by a strict lexical-semantic process (McRae et al., 1997). In fact, Hare et al. (2009, p.152) claim that “thematic role information is often described as an aspect of a verb’s argument structure, but its role in comprehension goes beyond strictly linguistic knowledge to reflect the comprehender’s understanding of how situations plausibly occur in the world.”

Other authors have focused on the activation of world knowledge at the sentence level. For example, Matsuki et al. (2011) examined how the combination of our common knowledge about instruments and actions can lead people to quickly process the incoming patient. To do so, participants were asked to read sentences such as the following:

Typical

- Donna used the hose to wash her filthy car.
- Donna used the shampoo to wash her filthy hair.

Atypical

(2c). Donna used the shampoo to wash her filthy car.
(2d). Donna used the hose to wash her filthy hair.

The critical manipulation concerned whether people can combine instruments (*hose* versus *shampoo*) and verbs (*wash*) to produce expectations for classes of patients (*car* and *hair*). It is crucial to note that in the atypical condition no selectional restriction is violated –sentences are atypical yet possible–, so that all sentences are equally acceptable from the point of view of lexical semantics. However, a typicality effect was found at the critical word (the patient of the non-finite verb) in first fixation durations and in first pass reading times, suggesting that event-based knowledge is quickly accessed and used during language comprehension.

## The present study

The present study aims to explore the effect of world knowledge on the prediction of incoming events in sentence processing by examining whether plausibility, as determined by patient-agent combinations, can influence reading times for the ensuing verb in the absence of selectional restriction violations. To the best of our knowledge, this hypothesis has not been tested before.

We used two experimental conditions: typical and atypical sentences. In the former, we chose good agents for a certain event (i.e., *journalist* is a good agent for *interview*). In the latter, we selected possible yet implausible agents (i.e., *terrorist* is a bad agent for *interview*). As we wanted to use a counterbalanced design, we built up quartets in which each agent was linked to each verb:

Typical

- A Pedro, un periodista lo entrevistó cuando estaba a punto de entrar en el portal.
  (Literal translation: To Pedro, a journalist interviewed him when he was about to enter his hallway).
- A Pedro, un terrorista lo secuestró cuando estaba a punto de entrar en el portal.
  (To Pedro, a terrorist kidnapped him when he was about to enter his hallway)

Atypical

(3c). A Pedro, un periodista lo secuestró cuando estaba a punto de entrar en el portal.
  (To Pedro, a journalist kidnapped him when he was about to enter his hallway).
(3d). A Pedro, un terrorista lo entrevistó cuando estaba a punto de entrar en el portal.
  (To Pedro, a terrorist interviewed him when he was about to enter his hallway).

All experimental sentences began with a topicalized PP that played the role of patient/recipient of the main verb. The PP was composed by the preposition *a* (*to*, in English), that is used with animate definite direct and indirect objects in Spanish, followed by a proper name (e.g., *Pedro*). Proper names were used because they only provide information about the gender of the participant. Combining biased patients and biased agents would facilitate the prediction of the verb, and we aimed to examine a subtler effect. The role of the patient/recipient was limited to provide some relevant syntactic information (e.g., the verb will be transitive or ditransitive) that could help predicting the upcoming verb. The patient/recipient was immediately followed by the agent and the event (verb).

We conducted an eye-tracking during reading experiment and predicted longer reading times at the critical word (the verb) in the atypical condition as compared to the typical condition due to the activation and rapid use of world knowledge information. Critically, if world knowledge information has a rapid effect on sentence comprehension, then one would expect the effects to emerge at the critical word in measures that reflect the early stages of processing. In contrast, if the effects of world knowledge information are delayed, then at the critical word one would expect no reliable effects or effects at measures that reflect later stages of processing.

### Method

#### Participants

Forty native speakers of Spanish (34 females) aged 18 to 58 (mean age 23.6), from the Universitat Rovira i Virgili took part in the eye-tracking experiment for academic credits. None had any diagnosed reading impairments, and all of them had normal or corrected-to-normal vision. All participants provided signed informed consent prior to the experiment. The study was approved by the Ethics Committee on Research into People, Society and the Environment (CEIPSA) of the Universitat Rovira i Virgili.

#### Materials

There were 32 quartets of experimental items, each of which appeared in the four conditions illustrated in (3). The stimuli were distributed across four Latin-square counterbalanced lists, such that each list contained exactly one condition from each of the 32 experimental items, and all conditions of all items were represented across the four lists. Sentences within a quartet were identical, except for the agent word (word 4) and the critical word (i.e., the verb, word 6). The agent word was the same in two conditions (in one condition, the agent preceded a typical verb, whereas in the other, it preceded an atypical verb) and the critical word was the same in two other conditions (in one condition, the verb was preceded by a typical agent, whereas in the other, it was preceded by an atypical agent).

Each experimental list contained 32 experimental sentences (8 per condition) mixed with 96 fillers which included a variety of different constructions. It is important to note that none of the atypical sentences presented a violation of selectional restrictions (all direct and indirect objects were [+ animate, +human], thus respecting the selectional restrictions of main verbs). The atypical sentences described unlikely, but possible situations. Yes-No comprehension questions appeared on 25% of the trials. These questions were never about critical words.

#### Procedure

Upon arrival participants were given information and consent forms. Readers’ heads were stabilized using a head and chin rest. Items were pseudo-randomised across four lists in a Latin-square design. A different order was presented to each participant. The experiment was conducted using an SR-Research EyeLink 1000 desk-mounted eye-tracker (SR Research: Mississauga, Canada) sampling at 1000 Hz. While viewing was binocular, eye-movements were recorded from the right eye. Sentences were displayed in Times New Roman 18 point on the screen with a maximum of one line per sentence. Following a successful calibration, participants completed five practice trials before the experiment began. Each trial began with a gaze trigger box, which appeared in the position of the first character of the text. When the gaze box had been successfully fixated, it was replaced by the full stimulus text. If the gaze trigger became inaccurate, the participant was recalibrated. Participants read each text silently and were asked to read the sentences at their natural speed and to answer a yes/no comprehension question for some of the sentences. The comprehension questions probed general understanding of the sentences. Participants had to answer the comprehension questions by clicking one of the two mouse buttons. They then read a total of 128 sentences, taking a break after reading the first 64 items. The experiment took approximately 40 min.

### Data Analysis

We report analysis for two regions of text. The critical region consisted of the critical verb, while the spillover region contained the following three words. The fixation data for these two regions were analysed according to three different eye-movement measures, which give a range of information about the time course of processing. First-pass reading times are calculated by summing the fixations in a region, between the time when the reader’s eye-gaze first enters the region from the left, to the time when the region is first exited to either the right or left. Regression path times (sometimes called go-past times) are the sum of fixations from the first entry into the region from the left, until that region is first exited to the right -in other words, the time taken for the reader to go past the region following the first forward saccade into the region. Note that regression path times always correspond to first-pass reading times if the region is first exited to the right. However, regression path times differ from first-pass reading times if the first exit from the region is a regression. In such cases, the regression path times include all fixations during that regression, plus any re-fixations on the critical region before the eye-gaze proceeds to subsequent regions. In addition to the two measures described above, we will report total reading times as a measure of delayed (or later) processing. Total reading times are the sum of all fixations within a region. Regions that were initially skipped during reading were treated as missing data in first-pass and regression path times, and regions that received no fixations at all were treated as missing data in total reading times.

Before the analyses, and as is common practise in eye-tracking while reading studies (e.g. Featherstone & Sturt 2010; Kwon & Sturt, 2016), fixations of less than 80 ms were incorporated into larger fixations within one character, and then any remaining fixations of less than 80 ms were deleted. Fixations longer than 1200 ms were also removed prior to analysis.

The analysis was conducted using linear mixed-effects models with crossed random effects for subjects and items (Baayen et al., 2008). All models were run using the lme4 package (Bates, 2005) in R (R Core Team 2018). Analysis was conducted on log-transformed reading times to minimise skew (see Vasishth & Nicenboim 2016). Models included sum coded (-0.5, 0.5) fixed main effect of ‘typicality’ (typical sentence vs. atypical sentence).

For all the models reported below, we followed the same three-step strategy: (1) We fit a model with a maximal random effects structure (Barr et al., 2013). This maximal model included by-subject and by-item random intercepts, and random slopes for the fixed effect and correlations between random slopes. If this model did not converge, (2) we removed the random correlation parameters and refit the model (see Barr et al., 2013). If the resulting model still did not converge, (3) we identified random slopes accounting for less than 1% of the variance of their associated random factors, then removed all such slopes simultaneously (Bates, Kliegl, Vasishth, & Baayen, 2015). In case of a singular fit, we removed random slopes with zero or near zero variance until a non-singular fit was obtained. The experimental sentences, as well as the full dataset and analysis code for the experiment reported here can be found at the last author’s OSF repository (https://osf.io/y95g2/).

## Results

Data from three participants were not included in the analysis because their error rate in answering the comprehension questions was higher than 15%. For the remaining participants, the overall accuracy to the comprehension questions was 95%, indicating that participants paid attention to the content of the sentences. Summaries of the reading time data are presented in Table 1. Note that although all models were run with log transformed reading times, the tables present raw reading times.

**Table 1:** Reading times in milliseconds for three eye-movement measures at the critical and spillover regions (SDs in parentheses).

	First pass reading time	Regression path time	Total reading time
*Critical region*			
Typical	342 (209)	508 (416)	671 (447)
Atypical	351 (216)	528 (389)	778 (539)
*Spillover region*			
Typical	481 (256)	656 (496)	962 (687)
Atypical	473 (270)	738 (549)	1037 (638)

At the critical region, first pass reading times revealed no significant differences between the two levels of the typicality factor (estimate = 0.022, SE = 0.030, *t* = 0.724, *p* = 0.474). In regression path times and total reading times there was a significant main effect of typicality (regression path times: estimate = 0.075, SE = 0.032, *t* = 2.306, *p* = 0.028; total reading times: estimate = 0.133, SE = 0.034, *t* = 3.855, *p* < 0.001). In both measures, reading times were longer in the atypical condition than in the typical condition (for regression path times, 528 ms vs. 508 ms; for total reading times, 778 vs. 670 ms).

At the spillover region, first pass reading times revealed no significant differences between the two levels of the typicality factor (estimate = -0.040, SE = 0.027, *t* = -1.49, *p* = 0.136). In regression path times and total reading times there was a significant main effect of typicality (regression path times: estimate = 0.094, SE = 0.030, *t* = 3.11, *p* = 0.006; total reading times: estimate = 0.082, SE = 0.025, *t* = 3.257, *p* = 0.003). In both measures, reading times were longer in the atypical condition than in the typical condition (for regression path times, 738 ms vs. 656 ms; for total reading times, 1037 vs. 962 ms).

## Discussion

The results of the experiment showed that at the critical region (i.e., the main verb), the main effect of typicality was reliable both at regression path time and at total reading time, with reading times being longer following atypical agents as compared to typical ones. At the spillover region the pattern of results was similar, in that the main effect of typicality was significant at regression path duration and total reading time, again with reading times being longer in the atypical condition as compared to the typical condition. The main effect of typicality was not significant in first pass reading times neither at the critical region nor at the spillover region. In sum, the effect of typicality showed up in the critical region in regression path times, a measure that indexes processing costs that occur in a particular region before subsequent material is fixated (i.e., before the eye-gaze proceeds to the right). That is, the effect of typicality emerged not only in a measure (i.e., total reading time) indicative of later processing, but in a measure that can be seen to reflect intermediate processes, possibly including processes that accompany the integration of the critical word with the preceding context.

The results of the present study clearly show that a minimal context, such as the subject/agent of a verb, can facilitate the processing of a verb when the subject is a good agent of the event denoted by the verb. The present findings are difficult to explain by a strictly lexically-based account, since there were no semantic nor selectional restrictions violations (all agents and patients/recipients had the [+ human] feature, satisfying the requirements of the verbs) in any of the sentences, and the same verbs and the same agents appeared in both conditions. The results strongly suggest that experience-based world knowledge is, thus, the source of the observed effects.

In line with previous proposals (Kuperberg, 2013; Paczynski & Kuperberg, 2012; Warren et al., 2015), we support the idea that selectional restrictions are, in fact, verb-related coarse-grained abstractions across world knowledge information. The violation of a fine-grained conceptual abstraction we have about a certain event (e.g., journalists are the typical agents of an interview) can result in an atypical yet possible situation (e.g., a terrorist interviewing someone); however, the violation of a coarse-grained abstraction (e.g., that the entity that interviews someone must be animate) normally results in a very anomalous sentence.

Nonetheless, it is also true that we found no such typicality effects in first pass reading times, contrasting with Matsuki et al. (2011). We think the neutrality of the patient/recipient (i.e., a proper name) in our study can account for this. Whereas in Matsuki et al. (2011) there were two important sources of conceptual information (biased instruments and verbs) that made it possible to quickly activate a certain event-based knowledge to predict the verb, the only relevant conceptual source of information in the present experiment was the agent, resulting in weaker predictions and slightly delayed effects (i.e., no effects in first pass reading times, but effects in regression path durations both in the critical and the spillover regions).

## Conclusions

The purpose of the present study was to test whether common world knowledge that is computed by combining a neutral patient/recipient (i.e., a proper name) and a conceptually biasing agent could lead people to predict the kind of upcoming verb in the sentence, thus facilitating its processing. We take our results as evidence supporting the crucial role of world knowledge during sentence comprehension, since the reported effects of typicality can only be explained by the activation and rapid influence of experience-based event knowledge. Two facts reinforce this interpretation: that all critical items within a quartet were identical except for the agent and verb, and that selectional restrictions were not violated in any of the atypical sentences. However, further research on the role of event knowledge in the prediction of upcoming verbs is needed to explore whether this source of information has earlier effects during sentence processing. Immediate effects of world knowledge in first pass reading times might emerge in sentences in which both the agent and, crucially, the patient/recipient preceding the verb provide more constraining information, thus increasing the predictive strength that might facilitate the processing of the incoming verb.

## Data Availability

The experimental sentences, as well as the full dataset for the experiment reported here can be found at the last author’s OSF repository (https://osf.io/y95g2/).

## Code Availability

The analysis code for the experiment reported here can be found at the last author’s OSF repository (https://osf.io/y95g2/).
